# Interlaboratory comparison of femur surface reconstruction from CT data compared to reference optical 3D scan

**DOI:** 10.1186/s12938-018-0461-0

**Published:** 2018-03-02

**Authors:** Ehsan Soodmand, Daniel Kluess, Patrick A. Varady, Robert Cichon, Michael Schwarze, Dominic Gehweiler, Frank Niemeyer, Dieter Pahr, Matthias Woiczinski

**Affiliations:** 1 Biomechanics and Implant Technology Research Laboratory, Department of Orthopaedics, University Medicine Rostock, Doberaner Strasse 142, 18057 Rostock, Germany; 2 Trauma Center Murnau Institute of Biomechanics, Professor-Küntscher-Str. 882418, Murnau am Staffelsee, Germany; 3 Chair of Mechanics and Robotics, University Duisburg-Essen, Lotharstrasse 1, 47057 Duisburg, Germany; 4 Laboratory for Biomechanics and Biomaterials of the Orthopaedic Clinic, Hannover Medical School, Anna-von-Borries-Strasse 1-7, 30625 Hannover, Germany; 5 Department of Trauma, Hand and Reconstructive Surgery, University Hospital Münster, Albert-Schweitzer-Campus 1, 48149 Münster, Germany; 6 Fraunhofer Research Institution for Large Structures in Production Engineering (IGP), Albert-Einstein-Str. 30, 18059 Rostock, Germany; 7 Institute of Lightweight Design and Structural Biomechanics, TU Vienna, Getreidemarkt 9, 1060 Vienna, Austria; 8 Department of Orthopedic Surgery, Physical Medicine and Rehabilitation, University Hospital of Munich (LMU), Marchioninistr. 15, 81377 Munich, Germany

**Keywords:** Accuracy assessment, Deviation analysis, Image-based model, Bone segmentation, Shape reconstruction, Medical imaging, Round robin test

## Abstract

**Background:**

The present study contrasts the accuracy of different reconstructed models with distinctive segmentation methods performed by various experts. Seven research groups reconstructed nine 3D models of one human femur based on an acquired CT image using their own computational methods. As a reference model for accuracy assessment, a 3D surface scan of the human femur was created using an optical measuring system. Prior to comparison, the femur was divided into four areas; “neck and greater trochanter”, “proximal metaphysis”, “diaphysis”, and “distal metaphysis”. The deviation analysis was carried out in GEOMAGIC studio v.2013 software.

**Results:**

The results revealed that the highest deviation errors occurred in “neck and greater trochanter” area and “proximal metaphysis” area with RMSE of 0.84 and 0.83 mm respectively.

**Conclusion:**

In conclusion, this study shows that the average deviation of reconstructed models prepared by experts with various methods, skills and software from the surface 3D scan is lower than 0.79 mm, which is not a significant discrepancy.

## Background

Over the past decade, the application of medical imaging technology in clinical diagnostics and therapy has expanded to diverse approaches such as evaluation of joint biomechanics, patient specific implant development and evaluation, statistical modelling, three-dimensional (3D) printing and rapid prototyping, computer-assisted surgery, and preoperative planning [1–4]. In order to examine the biomechanical behavior of human joints and tissues for all above-mentioned applications, subject specific image based finite element (FE) analysis has been used as the most commonly used method [5]. Extracting the bone geometry from medical images, generating an optimum FE mesh, assigning proper material properties, and defining actual boundary conditions are the main inputs for FE analysis [6], and therefore their accuracy affects the precision of the FE analysis result [7]. The file format for performing 3D analysis is different from medical image formats for computed topography (CT) to magnetic resonance imaging (MRI). Digital imaging and communication in medicine (DICOM) file formats must be converted to a format readable by CAD or finite element software for further 3D analyses, e.g. the stereolithography (STL) format. The first milestone of construction workflow of image-based biomechanical analysis is accurate segmentation (extracting desired bone geometry from medical image data) from source data [8–10]. Therefore, FE analysis for the aforementioned purposes requires accurate 3D shape representation of bones. This is fundamental to obtain segmented bone data as authentic as possible to the patient’s morphology. Since there are many commercial segmentation software packages and algorithms, The STL models segmented with commercially available software packages may have discrepancies compared to the actual bone, and the accuracy of the segmented bone could then vary based on the segmentation method and operator’s skills. Hence, the accuracy assessment of 3D reconstructed bone based on medical images has been recently investigated extensively [8, 11–25]. Yet, it is concerned that such segmented models do not represent the accuracy of the original bone in a FE model. Therefore, the purpose of this study is to investigate the effect of using different segmentation methodologies conducted by experts with different experiences and skills through a round robin test including seven biomechanics laboratories. The deviation of segmented bone models from the cadaver bone geometry obtained by optical 3D scanning was investigated and the discrepancies between reconstructed models from CT images and the model obtained from optical 3D scanning was quantified.

## Methods

**Fig. 1:**
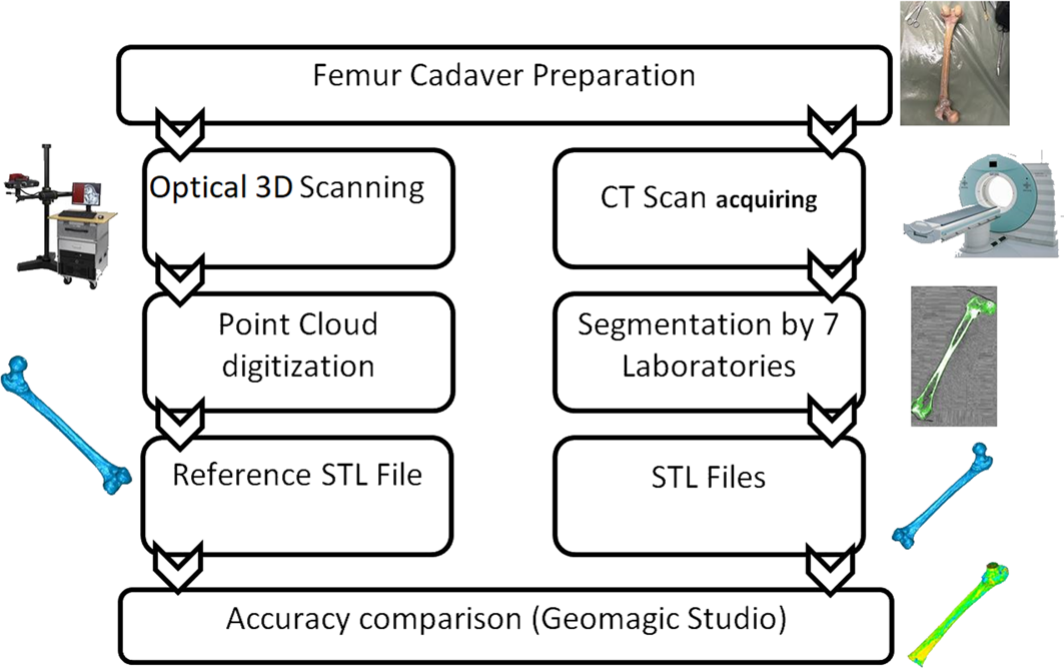
Flowchart of methodology. On the left column different steps of making reference model is shown including optical 3D scanning, point cloud digitization and creating STL files. Right column shows that the only CT scan image taken in the study was segmented by 7 laboratories to create 7 STL files

Seven different research groups were invited to participate in this interlaboratory comparison. The results of this research were anonymized and only the principal investigator had access to non-anonymized data. The laboratories were randomly numbered from 1 to 7. In case that more than one model was created by a laboratory, the first and second model of each laboratory was named by additional letters A and B, respectively. Figure 1 presents a flowchart showing the research methodology applied in this study. It briefly shows how the reference STL file and also different laboratories STL file were created.

### CT scan acquiring

The CT image of the right femur of a 58 year-old male cadaver was acquired from Trauma Center Murnau with a SOMATOM Definition AS + CT scanner (Siemens AG, Erlangen, Germany). This unique CT image was used by all seven laboratories for segmentation. Soft tissues were removed from the bone prior to the scan. The CT image was saved as DICOM with resolution of 0.29 × 0.29 mm and slice thickness of 0.6 mm for deviation analysis. To reduce partial volume effects, the femur was scanned in a water bath. In order to achieve a calibrated scan, a bone mineral density phantom was scanned in the same setup.

### Optical 3D digitization

**Fig. 2:**
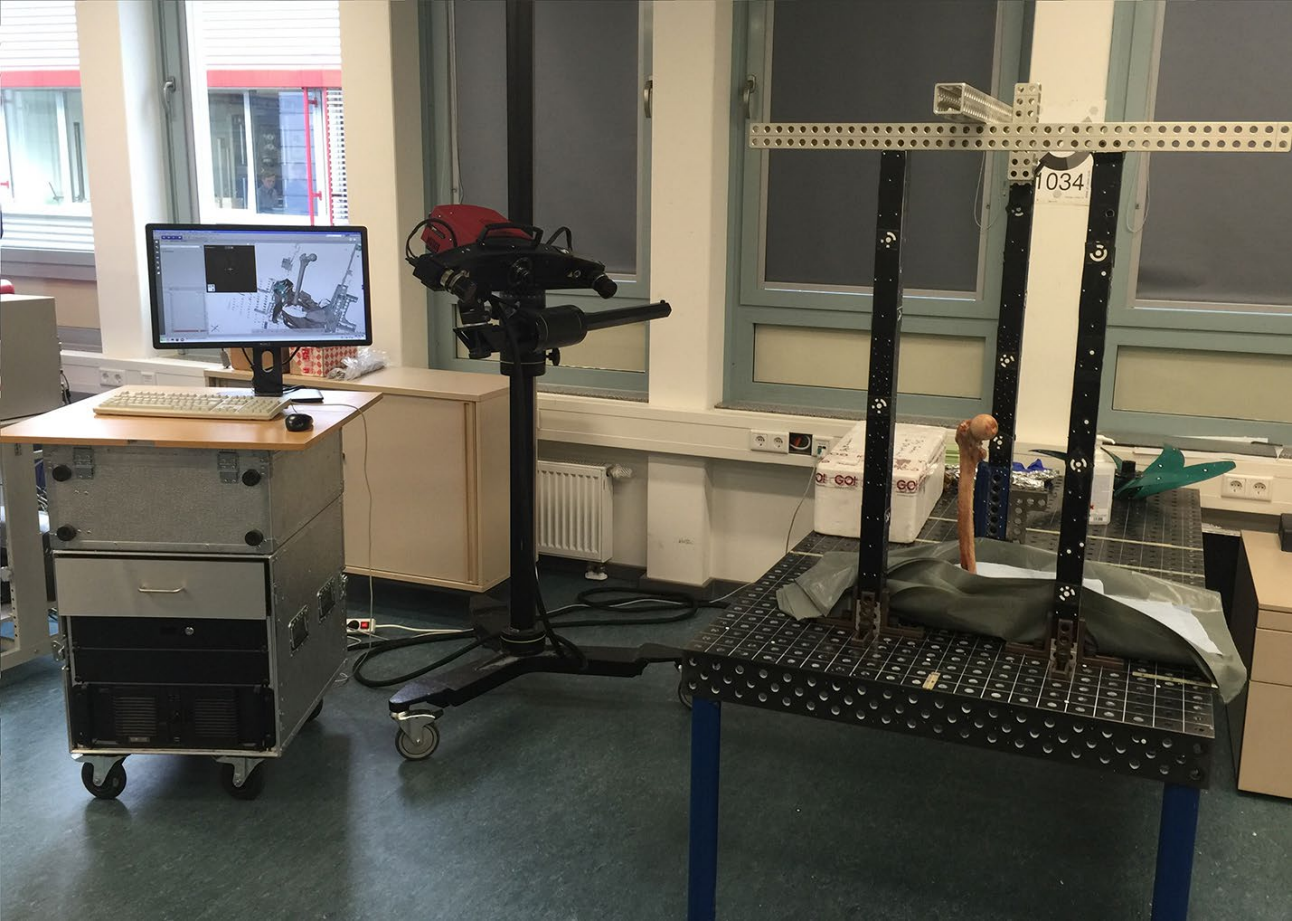
Optical 3D scanning setup at AGP Fraunhofer Institute in Rostock. The bone is located on the bench, scanning is performed by the optical scanner on the movable stand and controlled by the computer

The outer geometry of the femur was scanned using an optical measuring system at the Fraunhofer Application Centre of Large Structures in Production Engineering (AGP) in Rostock (Fig. 2). The ATOS series of industrial optical 3D scanners provide accurate scans with detailed resolution at high speeds (GOM-Gesellschaft für Optische Messtechnik mbH, Braunschweig, Germany). Instead of measuring single points, ATOS captures an object’s full surface geometry and primitives precisely in a dense point cloud or polygon mesh. The 3D scanner consists of two cameras and a projection system in which the projector projects special stripe light patterns on a surface of an object, being recognized by the cameras. Every scan produces a point cloud with up to 4 Billion points. Several scans from different points of view can be registered by special reference points with a defined diameter, which will be automatically detected by the software. Table 1 presents the specifications of the ATOS scanner for scanning the femur.

**Table 1:** Optical 3D scanner system specifications

Measuring field (xyz) 500	500 × 500 (mm^2^)
Distance between points	0.24 (mm)
Accuracy (probing/spacing/flatness)	MV500: 0.009/0.030/0.017 (mm)
Resolution	2048 × 2048 (4 megapixels)
Scan time	2.0 (s)
Dimensions	690 (W) × 220 (H) × 160 (D) (mm)

Structured Light Projection System GOM ATOS III

### Reconstruction of 3D models

DICOM files obtained from the CT scan were used to segment the surface of femurs by four different segmentation software packages: AMIRA^®^ (FEI Visualization Sciences Group, Oregon, USA), Mimics^®^ (Materialise N.V., Leuven, Belgium), YaDiv (Welfenlab, Leibniz Universität Hannover, Hannover, Germany) [26], and Fiji Life-Line [27]. The bony regions were labeled in all slices of the CT images based on the certain values of Hounsfield units (HU) for bones. The process of labelling of bony structures is a visual and subjective procedure in which a primary HU value for bones was selected from the literature which is around 200–250 up to 3000 [28–31]. The automated segmentation of the bone started by thresholds of HU and was followed by manually editing the slices to obtain more accurate surfaces [28–31]. A triangulated surface of the femurs was created with the segmentation software using a semi-automatic method. Removal of the holes and sharp edges which were formed due to semi-automated segmentation was implemented in the above mentioned software [31–34]. Software information, methods and duration of the segmentation process for each model are tabulated in Table 2. The reconstruction process of CT image was performed by researchers with 3–5 years of experience in segmentation.

**Table 2:** Segmentation information such as segmentation software, time taken for segmentation, and segmentation method for each participant

	Segmentation software	Time (min)	Segmentation method
Laboratory 1	Mimics 18	480	Semi-automatic + manuel editing (3-Matic v.10)
Laboratory 2A	AMIRA^®^ v.5.3.3	480	Semi-automatic + manuel editing (MeshLab 1.3.4)
Laboratory 2B	YaDiv 1.0 beta 5	480	Semi-automatic + manuel editing (MeshLab 1.3.4)
Laboratory 3	AMIRA^®^ v.5.4.1	600	Semi-automatic + manuel editing
Laboratory 4	AMIRA^®^ v.6	330	Semi-automatic + manuel editing (Geomagic Studio v.2012)
Laboratory 5	AMIRA^®^ v.5.6	480	Semi-automatic + manuel editing (Geomagic Studio v.2012)
Laboratory 6	Fiji-Medtool v.4.0	85	Full-automatic + manual editing
Laboratory 7A	AMIRA^®^ v.5.4.1	270	Semi-automatic + manuel editing (Geomagic Studio v.2013)
Laboratory 7B	Mimics v.17	340	Semi-automatic + manuel editing (3-Matic v.9)

### Deviation analysis

**Fig. 3:**
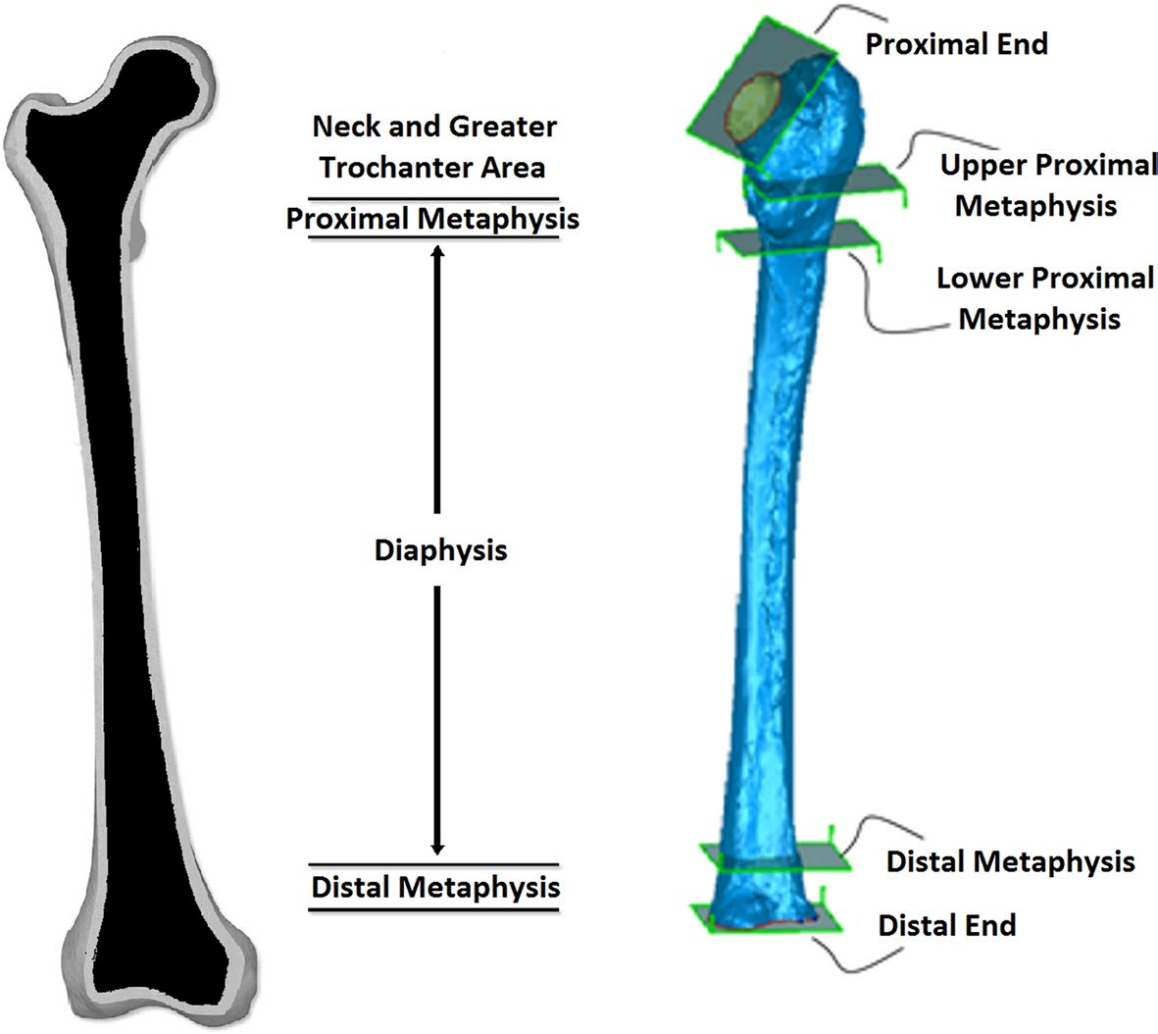
Five predefined planes for splitting femur into 4 pieces to perform the deviation analysis

Stereolithography (STL) files were collected from all project partners and imported into GEOMAGIC studio v.2013 (Raindrop Geomagic, NC, USA) for deviation analysis. Thereby, the researcher conducting the analysis was blinded towards the participant’s identity in order to avoid bias. Prior to comparison, the femur was divided into 4 areas: “neck and greater trochanter” area, “diaphysis”, “proximal metaphysis” and “distal metaphysis”. Five different planes were defined in global coordinates to divide all models into above-mentioned areas. Proximal end, upper proximal metaphysis, lower proximal metaphysis, distal metaphysis and distal end were the predefined planes for splitting the models into four aforementioned parts [35]. Figure 3 illustrates the predefined cutting planes of the femur. The Neck area includes “neck and greater trochanter” and the “proximal metaphysis” contains the area of lesser trochanter. The “diaphysis” defined as long bone known as the femur shaft and the last part excludes the epiphysis named “distal metaphysis”.

## Results

**Fig. 4:**
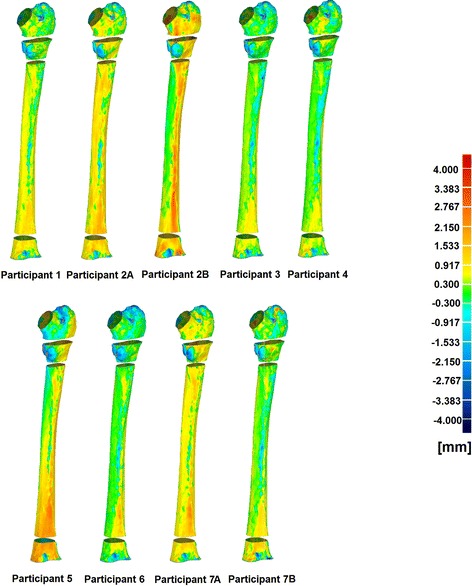
surface geometries comparison of 9 reconstructed models with the optical 3D scanned surface model. The red surface areas show overestimating of the reference model and blue areas indicate underestimation

**Fig. 5:**
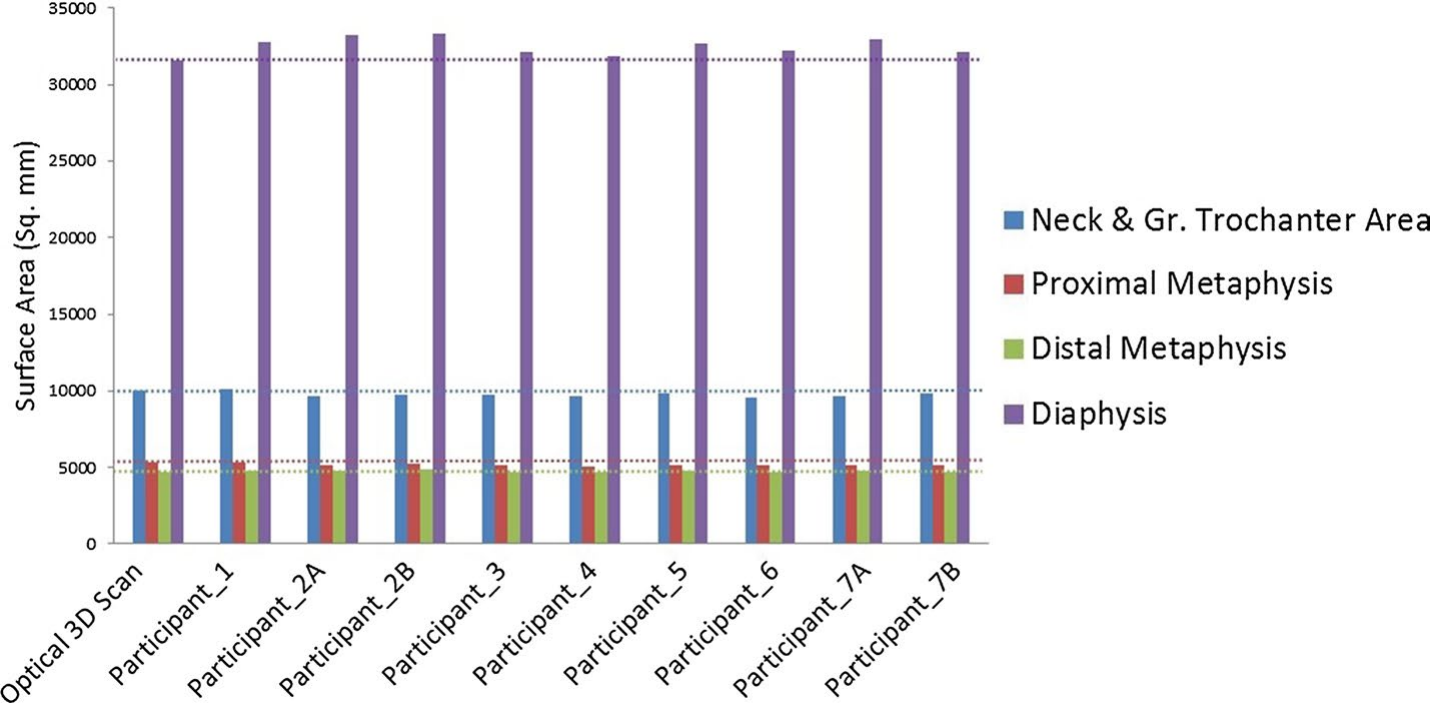
Surface area of 4 parts (neck and great trochanter area, proximal metaphysis, distal metaphysis and diaphysis) of the femur obtained from optical 3D scan (reference STL file) as well as 7 participant laboratories

Seven laboratories prepared nine reconstructed models out of one single human femur CT scan. The outer surface of the femur was scanned with high point resolution using an optical 3D scanner. The results of 3D deviation analysis for the four femoral parts were processed in GEOMAGIC studio and compared to the other models. Table 3 presents the average deviation values of the 9 different segmented models for all four predefined areas of the femur. The highest deviation was observed in “neck and greater trochanter” area with RMSE of 0.84. The negative values for the estimated percentage error of the surface areas represent the deviation of the underestimated areas and the positive values show the overestimated areas (see Table 3). Root means square error (RMSE) was used as a standard statistical metric for comparison and evaluation of simulation models performance [36, 37]. Figure 4 also illustrates the visual deviation using color-coded map to show the differences of each model compared to the bone optical 3D scan. Figure 5 illustrates the estimated surface areas of the nine segmented models and the bone optical 3D scan. “Diaphysis” and “neck and greater trochanter” areas have the largest percentage errors of outer surface area with 2.92 and 2.57% respectively. This figure indicates that the outer surface areas of the reconstructed models are not exceedingly different from the reference model.

**Table 3:** Average deviation of four different parts of femur

	Average deviation positive (mm)	Average deviation negative (mm)	Standard deviation (mm)	RMSE (mm)	Average percentage errors of surface area (%)
Neck and greater trochanter area	0.48	− 0.72	0.78	0.84	− 2.57
Proximal metaphysis	0.61	− 0.78	0.78	0.83	− 2.06
Diaphysis	0.63	− 0.18	0.41	0.69	2.92
Distal metaphysis	0.66	− 0.50	0.56	0.73	0.86

## Discussion

Finite element models are commonly used based on specific geometrical characteristics extracted from medical imaging data. This study presents a deviation analysis to evaluate different segmentation methods based on CT scan compared to optical 3D surface scan of the same bone. Thereby, the reconstruction results of seven different biomechanics laboratories were compared to evaluate how human skills, methods of segmentation, and different software packages can cause imprecision in image-based reconstructed models.

This study investigated a variety of conditions, which may have influenced the accuracy of the segmentation process. The segmentations of “neck and greater trochanter” area and “proximal metaphysis” showed the greatest deviations with RMSE of 0.84 and 0.83 mm respectively (Table 3). Thevenot et al. [38] reported the accuracy of a novel method for automatically reconstructed 3D model from 2D hip radiograph and Verim et al. [39] evaluated the reconstructed proximal femur from different images of different devices. They both found that the greatest error happened in the trochanter area which is in good agreement with our results. Vaananen et al. [40] assessed the 3D shape of proximal femur using two different methods; shape template and bone mineral density image. They also found out that the maximum discrepancies are in trochanter area, ranging from 0.7 to 2.6 mm. Our results also showed the similar range of discrepancies. Schumann et al. [41] used clinically relevant morphometric parameters measurement of the proximal femur to examine the accuracy of their reconstructed method. In their study, the highest average deviations were also observed in trochanter area. Rathnayaka et al. [42] conducted a study to compare the accuracy of MRI and CT reconstructed 3D models where they also estimated the highest deviations was observed in the “neck and greater trochanter” region. The highest deviations, usually observed in the “neck and greater trochanter” area, are probably due to geometrical complications exist in this area. In the current study, the highest estimated discrepancy from the reconstructed models is 0.79 mm. The previous studies of Glaude et al. [11, 43] on accuracy assessment of reconstructed models based on medical images, suggest that the mean 3D deviation of reconstructed models should be in the range of 1 mm. Since clinical hip fractures commonly occur in the neck area [38], more accuracy in reconstruction of this area is required to have more precise FE analysis results. Furthermore, as observed in Fig. 5, there was no outlier in the accuracy assessment comparison, and all the reconstructed 3D models have similar range of deviations. However, if peak discrepancies are observed, they can be simply disregarded because they are local. The outer surface areas of the reconstructed models providing the surface meshes for FE analysis were also estimated in this study. Highest errors of the outer surface area were observed in “diaphysis” and “neck and greater trochanter” regions. This is also illustrated in Fig. 4 using color-coded map to show that “diaphysis” and “neck and greater trochanter” regions have the highest surface discrepancies compare to the real bone 3D optical scan. Therefore, these two regions are the most critical regions for reconstruction of 3D models based on medical images and should be processed carefully. The results also suggest, that the quality of the image segmentation is rather independent of reconstruction processing software. The differences observed in segmentation times can be associated with either individual investigator speed of segmentation or the usability of the software. For future works, the effect of the negligible discrepancies on the FE analysis results will be examined using a controlled load case in an experimental setup.

## Conclusion

This study shows that the average deviation of CT based models, prepared by experts with different skills using various software packages, from a bone surface scan is very low. This reveals that the effect of human expertise and use of different software packages and corresponding methodologies have a negligible effect on the accuracy of the reconstruction procedure from medical images. Therefore, image-based reconstructed models are reliable to use in FE models for clinical applications.
